# Oral side effects of dermatologic therapies: A systematic review and meta-analysis

**DOI:** 10.6026/973206300212833

**Published:** 2025-08-31

**Authors:** Reem M. Alqahtani, M Alsarrah Ahmad Farhan, Saud M. Almutiari, A. Althuwayb Fajr Ehab, F. Alanazi Abdulmohsen Abdulkarim, Abdalwhab Zwiri, Mohammed Ghazi Sghaireen, Mohammad Khursheed Alam

**Affiliations:** 1 Department of Dermatology, College of Medicine, Jouf University, Sakaka, Saudi Arabia; 2 Department of Prosthetic Dentistry, College of Dentistry, Jouf University, Sakaka, Kingdom of Saudi Arabia; 3 Department of Preventive Dentistry, College of Dentistry, Jouf University, Sakaka, Saudi Arabia; 4 Department of Dental Research Cell, Saveetha Institute of Medical and Technical Sciences, Saveetha Dental College and Hospitals, Chennai, India; 5 Department of Public Health, Daffodil International University, Dhaka 1207, Bangladesh, India

**Keywords:** Dermatologic agents, oral manifestations, drug-related side effects, adverse reactions, systematic review, meta-analysis

## Abstract

Dermatologic treatments can produce clinically significant oral side effects that remain poorly quantified. A significant association between dermatologic therapies and oral complications (ES=1.55, 95% CI: 0.94-2.17, p<0.001) is observed, despite high heterogeneity (I^2^=96%). Retinoids were predominantly associated with cheilitis and xerostomia, immunosuppressants with mucositis and ulcerations, and JAK inhibitors with opportunistic infections. Thus, data shows the necessity of routine oral assessments and preventive strategies to reduce therapy-related morbidity.

## Background:

Dermatologic therapies, including topical and systemic medications, are widely used to treat various skin conditions such as psoriasis, eczema and acne. While these treatments are effective in managing dermatologic diseases, they can also lead to unintended side effects, including oral manifestations [1]. Oral side effects, such as xerostomia, stomatitis and lichenoid reactions, are increasingly reported but remain understudied in systematic reviews [2]. Understanding these adverse effects is crucial for clinicians to optimize patient care and minimize treatment-related complications. The pathophysiology of oral side effects from dermatologic therapies varies depending on the drug class. Retinoids, immunosuppressants and biologics are known to disrupt oral mucosal integrity, leading to ulcerations and dysgeusia [3]. For instance, isotretinoin, commonly prescribed for acne, is associated with cheilitis and mucosal dryness [4]. Similarly, methotrexate, used in psoriasis, might cause oral mucositis due to its cytotoxic effects [5]. Despite these observations, a comprehensive synthesis of evidence on oral adverse effects is lacking. Previous studies have explored dermatologic drug toxicities, but most focus on systemic or cutaneous reactions rather than oral complications [6]. Therefore, it is of interest to consolidate available evidence, evaluate methodological quality, and provide clinically relevant recommendations for managing oral side effects of dermatologic therapies.

## Review:

This systematic review and meta-analysis followed PRISMA guidelines. A comprehensive search was conducted across databases and included studies reporting about oral side effects of dermatologic therapies.

## Search strategy and database queries:

The search strategy was designed using MeSH terms and keywords related to dermatologic therapies and oral side effects. Filters were applied to include human studies published in English between 2000 and 2024. Boolean operators and syntax modifiers were used to refine results across databases (Table 1 - see PDF). Manual searches of reference lists from included studies and relevant reviews were performed to identify additional articles. Conflicts in study selection were resolved through discussion between two reviewers, with a third reviewer consulted if consensus was not reached.

## PICO based eligibility criteria:

The PICO framework guided study selection; ensuring only relevant studies with reported oral side effects were included. Exclusions comprised non-therapeutic studies, animal research and publications lacking outcome data (Table 2 - see PDF).

## Data extraction process:

Two reviewers independently extracted data on study characteristics, interventions and outcomes using a standardized form. Discrepancies were resolved via consensus. Extracted data included sample size, adverse event rates and follow-up duration (Table 3 - see PDF).

## Risk of bias and publication bias evaluation:

The ROB 2 tool assessed bias in RCTs [7], while ROBINS-I evaluated non-randomized studies [8]. Funnel plots and Egger's test detected publication bias, with asymmetry indicating potential bias [9].

## Statistical analysis:

Meta-analysis was performed using RevMan 5.4, with pooled prevalence estimates calculated via random-effects models. Heterogeneity was assessed using I^2^ statistics, with subgroup analyses for drug classes and study designs.

## Results:

## Process for study selection:

The systematic review began with 987 records identified across four databases: PubMed (121), Embase (400), Cochrane Library (110) and Web of Science (356). After removing 756 duplicate records, 231 studies underwent title/abstract screening. Of these, 120 were excluded and full-text retrieval was attempted for 111 reports. Ultimately, 19 studies were assessed for eligibility, with 9 excluded due to unmet criteria (*e.g.*, irrelevant outcomes, lack of oral side effect data) [10, 11, 12, 13, 14, 15, 16, 17-18], leaving 10 studies for inclusion in the final review [19, 20, 21, 22, 23, 24, 25, 26, 27- 28]. This process followed PRISMA guidelines to ensure methodological rigor and minimize bias (Figure 1 - see PDF). The key findings highlighted common oral complications such as cheilitis (isotretinoin), mucositis (methotrexate) and infections (JAK inhibitors). Adverse event rates varied by drug class, with systemic therapies (*e.g.*, retinoids, immunosuppressants) showing higher oral toxicity (Table 4 - see PDF). The systematic evaluation of 10 studies revealed consistent patterns of oral adverse effects associated with various dermatologic therapies, highlighting both common and drug-specific complications. Retinoids, particularly isotretinoin, demonstrated the highest frequency of oral side effects, with cheilitis occurring in 75% of treated patients and xerostomia in 50%. These effects were dose-dependent and often required adjunctive therapies (*e.g.*, moisturizing agents) for management [20, 21, 28]. Liarozole, another retinoid, was associated with dry mouth (35%) and taste disturbances (15%), suggesting a class-wide effect on salivary gland function and mucosal integrity [25]. Immunosuppressants, including methotrexate and cyclophosphamide, were linked to oral mucositis (15-25%) and ulcerations, with cyclophosphamide showing a higher risk (25%) compared to methotrexate (15%) [23, 27]. These complications were attributed to the drugs' cytotoxic effects on rapidly dividing mucosal cells, emphasizing the need for prophylactic measures (*e.g.*, folate supplementation for methotrexate) [24]. JAK-STAT inhibitors (*e.g.*, tofacitinib) were associated with opportunistic infections, notably oral candidiasis (10-20%), due to their immunosuppressive properties [19, 26]. This risk underscores the importance of fungal prophylaxis in susceptible patients. Topical therapies, such as dapsone gel, rarely caused systemic effects like methemoglobinemia (<1%), but oral manifestations were indirect and tied to systemic absorption [22].

## Comprehensive evaluation of the risk of bias in included studies:

## Risk of bias:

The ROB-2 evaluation of randomized trials demonstrated that four studies [20, 25, 27, 28] had low risk of bias, whereas Sharquie *et al.* (2013) [21] raised concerns due to deviations from intended interventions (D2) and selective reporting (D5). In contrast, the ROBINS-E assessment of non-randomized studies revealed that three studies [22, 24, 26] had low risk of bias across all domains, while Yan *et al.* (2024) [23] was rated high risk due to confounding (D1) and selection bias (D3). Wong *et al.* (2011) [19] showed a moderate risk, primarily due to unaddressed confounding. These results highlighted the methodological rigor of RCTs compared to observational studies, with the former more susceptible to confounding and selection biases (Figure 2, 3 - see PDF).

## Publication bias:

The funnel plot displays effect sizes (ranging from -2.00 to 5.00) against their standard errors (0.10-0.70), showing symmetrical distribution around the combined effect size (CES), suggesting minimal publication bias (Figure 4 - see PDF). The meta-regression results indicated no significant association between study characteristics and effect sizes (intercept: β=1.42, p=0.730; slope: β=1.24, p>0.05), with wide confidence intervals (intercept 95% CI: -7.53 to 10.37; slope 95% CI: -0.12 to 2.60) reflecting substantial heterogeneity (Table 5 - see PDF). The adjusted CES and imputed data points further confirm robustness to outliers [29, 30].

## Meta-analysis findings:

## Forest plot:

The forest plot presented the effect sizes and 95% confidence intervals (CIs) of 10 included studies, with weights reflecting their contribution to the pooled analysis. Studies such as Yan *et al.* (2024) (ES=4.50, 95% CI: 3.37-5.63) [23] and Wong *et al.* (2011) (ES=3.20, 95% CI: 2.26-4.14) [19] showed the largest effect sizes, while Al-Salama & Deeks (2017) (ES=0.10, 95% CI: -0.58-0.78) [22] and Herane *et al.* (2009) (ES=0.30, 95% CI: -0.10-0.70) [28] demonstrated minimal effects. The weights, ranging from 7.76% to 10.84%, were relatively balanced across studies. The variability in effect sizes and CIs suggested clinical and methodological heterogeneity, which might warrant subgroup analyses. Notably, all CIs except Al-Salama & Deeks (2017) [22] excluded the null value (ES=0), indicating statistically significant effects for most interventions (Figure 5 - see PDF).

## Heterogeneity assessment:

The random-effects meta-analysis of 10 studies revealed a moderate pooled effect size (correlation = 0.40, 95% CI: 0.71-2.52), indicating a statistically significant association between dermatologic therapies and oral adverse effects (two-tailed *p* < 0.001). However, the prediction interval (-0.35 to 3.58) and substantial heterogeneity (I^2^ = 96.01%, *p* < 0.001) suggested high variability in effect sizes across studies, likely due to differences in drug classes, study designs, or patient populations. The tau-squared value (0.59) further confirmed this variability, implying that true effects might differ significantly between contexts. Despite heterogeneity, the Z-value (4.04, *p*<0.001) supports the robustness of the overall association. These findings underscore the need for cautious interpretation and subgroup analyses to address heterogeneity [31] (Table 6 - see PDF).

## Subgroup analysis:

The subgroup meta-analysis revealed distinct patterns of oral adverse effects across different drug classes. Retinoids (Group A) showed a moderate pooled effect size (ES=1.17, 95% CI: -0.30-2.63) with extremely high heterogeneity (I^2^=97.95%), suggesting variable outcomes across studies. Immunosuppressants (Group B) demonstrated the strongest association (ES=1.98, 95% CI: 3.53-7.48), though with similarly high heterogeneity (I^2^=96.53%). JAK inhibitors and others (Group C) had an intermediate effect (ES=1.93, 95% CI: -0.18-4.03) with slightly lower but still substantial heterogeneity (I^2^=88.54%). The overall combined effect size was significant (ES=1.55, 95% CI: 0.94-2.17, p<0.001), confirming dermatologic therapies' association with oral adverse effects. However, the between-subgroup differences were not statistically significant (p=0.404), indicating that while effect sizes varied numerically, these differences might be due to within-group variability rather than true class-specific effects. The extremely wide prediction intervals, particularly for immunosuppressants (PI: -6.58-10.53), highlight the clinical unpredictability of these outcomes (Figure 6 - see PDF and Table 7 - see PDF). This subgroup analysis stratified studies by design to examine methodological influences on reported oral adverse effects. RCTs (Group A) demonstrated a moderate pooled effect size (ES=1.23, 95% CI: 0.23-2.24) with extremely high heterogeneity (I^2^=97.28%), reflecting variability in interventions and populations despite rigorous designs. Reviews (Group B) showed comparable effects (ES=1.15, 95% CI: -0.92-3.22) but slightly lower heterogeneity (I^2^=91.74%), while observational/case reports (Group C) had the largest point estimate (ES=3.83, 95% CI: -4.42-12.09), though with wide confidence intervals crossing the null value and substantial uncertainty (I^2^=73.22%). The overall combined effect (ES=1.99, 95% CI: 0.05-3.93) remained significant (p<0.001), but the prediction interval (-1.29-5.27) and extreme heterogeneity (I^2^=96.01%) suggested true effects might vary substantially in different contexts. Notably, the inflated effect sizes in observational studies likely reflect detection bias or confounding inherent to their designs (Figure 7 - see PDF).

## Discussion:

This systematic review and meta-analysis provide compelling evidence that dermatologic therapies are significantly associated with oral adverse effects, with a pooled effect size of 1.55 (95% CI: 0.94-2.17, p<0.001). The findings substantiate and extend previous research in this field while revealing important nuances in drug-specific toxicity profiles. The pronounced oral effects of retinoids, particularly isotretinoin, were remarkably consistent across studies. The current study's finding of 75% cheilitis incidence aligns precisely with the clinical observations of Elad *et al.* (2019), who noted that nearly all patients on systemic retinoids developed some degree of lip inflammation [2]. The pathophysiological basis for this class effect appears rooted in retinoids' ability to inhibit sebaceous gland function and alter epithelial differentiation, leading to mucosal dryness and fragility [1, 3]. Interestingly, while the current study found similar xerostomia rates (50%) to previous reports, the severity appeared less pronounced than in studies focusing on elderly populations, suggesting age might modify this adverse effect [8]. For immunosuppressants, currently observed ulcer rates (15-25%) were notably lower than the 30-40% reported by López-Pintor *et al.* (2015) [6]. This discrepancy likely reflects current analysis's exclusion of high-dose chemotherapy regimens and inclusion of newer, more targeted agents. The temporal trend toward reduced toxicity is encouraging and might reflect improved dosing protocols and prophylactic measures, such as folate supplementation for methotrexate patients [5]. However, the persistence of oral complications even with modern regimens underscores the need for continued vigilance.

The 10-20% incidence of oral candidiasis with JAK inhibitors mirrors Lacouture's (2018) systematic review [3], but current analysis revealed this risk emerges earlier in treatment (typically within 3 months) than previously recognized. This temporal pattern suggested that fungal prophylaxis might be most beneficial during initial therapy. The mechanistic basis likely involves both broad immunosuppression and specific inhibition of JAK-STAT pathways crucial for mucosal immunity [9]. The extraordinary heterogeneity (I^2^=96.01%) in the current analysis reflected fundamental challenges in studying dermatologic toxicities. Unlike prior meta-analyses that focused on single drug classes [7], the current study's inclusive approach captured the full spectrum of therapeutic agents, inevitably introducing variability. The lack of standardized toxicity grading scales across studies further compounded this issue. Nevertheless, the consistency of current study's key findings across subgroups supports their validity. However, the current study's failure to detect significant between-class differences (p=0.404) contrasts with Zaghloul *et al.*'s (2005) conclusion that retinoids are uniquely mucotoxic [4]. This discrepancy likes stems from the inclusion of newer targeted therapies in the current analysis that exhibit different toxicity profiles than traditional systemic agents. The evolving dermatologic pharmacopeia might be reducing historical disparities in oral adverse effects between drug classes. Several clinical implications emerge: baseline oral examinations should be routine before initiating dermatologic therapies, retinoid patients might benefit from prophylactic lip care regimens, JAK inhibitor recipients might need antifungal prophylaxis during early treatment and oral symptoms should be monitored regardless of drug class, as all showed significant effects. These findings highlighted the importance of interdisciplinary collaboration between dermatologists and oral medicine specialists to optimize patient care. Future studies should employ standardized oral toxicity metrics and longer follow-up to better characterize the natural history of these adverse effects.

## Limitations of the study:

High heterogeneity (I^2^>90%) limited the generalizability of pooled estimates, despite subgroup analyses. Second, small sample sizes in case reports (*e.g.*, Wong *et al.* *n*=1) inflated effect sizes. Third, publication bias could not be fully ruled out, though funnel plots appeared symmetrical. Finally, inconsistent reporting of adverse event severity precluded dose-response analyses.

## Future directions:

Future research should prioritize prospective RCTs with standardized oral toxicity scales (*e.g.*, WHO mucositis criteria) to reduce heterogeneity. Mechanistic studies exploring drug-mucosa interactions (*e.g.*, retinoid effects on salivary glands) could identify preventive strategies. Additionally, patient-reported outcomes should be incorporated to assess quality-of-life impacts overlooked in current literature.

## Conclusion:

Dermatologic therapies, particularly retinoids and immunosuppressants, significantly increase the risk of oral adverse effects, necessitating baseline oral assessments and prophylactic measures. Despite variability across drug classes, the consistency of effects underscores the need for multidisciplinary monitoring by dermatologists and dentists to mitigate complications.
